# Long‐Term Follow‐Up of Patients with Mass Social Media‐Induced Illness Presenting with Functional Tic‐like Behaviors

**DOI:** 10.1002/mdc3.70384

**Published:** 2025-10-08

**Authors:** L. Kathrin Hartung, Simon Schmitt, Carolin Fremer, Carolin S. Klages, Natalia Szejko, Kirsten R. Müller‐Vahl

**Affiliations:** ^1^ Clinic of Psychiatry, Social Psychiatry and Psychotherapy, Hannover Medical School Germany; ^2^ Department of Experimental and Clinical Pharmacology Center for Preclinical Research and Technology CEPT, Medical University of Warsaw Warsaw Poland

**Keywords:** functional movement disorder, Tourette's syndrome, functional tic‐like behaviors, prognosis, long‐term follow‐up

## Abstract

**Background:**

Only little is known about the prognosis of functional tic‐like behaviors (FTLB), especially in the subgroup of patients with mass social media‐induced illness (MSMI‐FTLB).

**Objectives:**

To provide data of long‐term follow‐up (FU) of a carefully characterized group of patients with MSMI‐FTLB to identify influencing factors such as treatment, exposure to social media, and comorbidities.

**Methods:**

At FU (mean = 26.3 (range = 11–51) months after baseline), 30 patients (mean age = 22.5 years, n = 18 (60%) female) underwent an extensive semi‐structured interview.

**Results:**

The majority of patients reported symptom improvement (n = 19, 63%) or complete remission (n = 7, 23%) of MSMI‐FTLB, while only a minority reported no change (n = 4, 13%), and none worsening of symptoms. Factors associated with better prognosis were younger age, male sex, diagnosis early after disease onset, less lifetime psychiatric comorbidities, absence of depression, and discontinuation of secondary gain, while diagnostic acceptance or changes in daily social media time had no influence on prognosis. Of n = 26 patients (87%) receiving psychotherapy, n = 15 (58%) rated it as “helpful,” but neither the presence of therapy nor the type of therapeutic approach was associated with significant differences in symptom improvement. At FU, n = 13 (43%) patients received medication, which most (n = 11, 85%) felt ineffective. N = 11 patients (36.7%) indicated that simply knowing the correct diagnosis was helpful in terms of symptom improvement.

**Conclusions:**

In line with previous reports in FTLB, patients with MSMI‐FTLB also have a good prognosis overall, especially in the case of younger age, male sex, early diagnosis, discontinuation of secondary gain, less psychiatric comorbidities, and absence of depression.

Functional movement disorders (FMD), defined by abnormal, involuntary movements without an identifiable neurological cause, have gained significant attention due to their complex etiology and challenging treatment.^1^ One type of FMD, functional tic‐like behaviors (FTLB), mimic Tourette's syndrome (TS) and are presenting with movements and vocalizations that resemble tics. Compared to TS, however, significant differences exist including later age of onset (≥12 years), rapid onset and progression of symptoms, and distinct phenomenological features such as higher prevalence of complex compared to simple tic‐like behaviors, occurrence of offensive words or statements, and a pronounced context dependency.^2^

Since 2019, the incidence of FTLB has notably increased, particularly among young people, sparking hypotheses that social and pandemic‐related stressors may play an important role.^3^, ^4^, ^5^, ^6^, ^7^ Consistent with observations of disease modeling,^8^ our team observed that many patients with FTLB seen in our outpatient clinic presented with symptoms closely resembling those of the influencer Jan Zimmermann, a popular German YouTuber, showing that social contagion is an important mechanism behind the development of FTLB.^5^ Our cohort met the criteria for mass sociogenic illness (MSI), which, in line with the established literature, are characterized by the rapid spread of symptoms within socially connected groups, the absence of an identifiable organic cause, and a functional rather than structural neurological pattern atypical for TS. Given that transmission occurred exclusively via social media rather than direct interpersonal contact, we suggested the new and more specific term “mass social media‐induced illness” (MSMI).^3^, ^5^

Over the last few years, several research groups also confirmed the important contribution of exposure to social media content showing individuals with apparent tics as a risk factor for FTLB.^4^, ^6^, ^7^, ^9^, ^10^ Furthermore, underlying psychological and physiological mechanisms such as emotion processing, attention, and interoception,^11^, ^12^ pre‐existing psychiatric illnesses including anxiety and depression, exposure to stressors as well as social and intergroup dynamics^13^ have been identified as relevant factors with regard to the development of FTLB.^12^, ^14^ The association between stress and social isolation became evident during the COVID‐19 pandemic, where disruptions in routine and heightened psychological distress paralleled an increase in FTLB incidence.^7^, ^15^, ^16^, ^17^, ^18^ Thus, similarly to other functional neurological disorders (FND),^19^ a biopsychosocial perspective is crucial for a comprehensive understanding of FTLB.

In addition to identifying predisposing and triggering factors, understanding the course of FTLB is crucial to better understand the pathogenesis in order to better inform patients regarding prognosis and to develop effective treatments strategies. In FND, prognosis varies widely, with some individuals showing improvement within short time periods and others experiencing persistent or even worsening of symptoms, or change from one to another type of FND.^20^ Key factors positively influencing prognosis include acceptance of the diagnosis and therapeutic adherence, while coexisting functional neurological symptoms have a detrimental effect.^21^, ^22^, ^23^ Studies on the prognosis of FTLB indicate that patients who engage in cognitive behavioral therapy or other psychotherapeutic interventions tailored to treat the patients’ anxiety or depression often experience a reduction in symptom severity, suggesting potential transdiagnostic etiological factors.^7^, ^23^, ^24^, ^25^ In addition to therapeutic approaches, the involvement and perceptions of family and friends play a crucial role in the course of symptoms and treatment outcomes.^26^ Furthermore, younger age appears to be associated with better prognosis, which may be explained by lower risk of chronicity, higher adaptive capacities, and potentially increased social support.^25^ The extent to which these variables impact long‐term outcomes, however, remains uncertain.

This present study investigated, for the first time, the long‐term prognosis of a specific type of FTLB, MSMI‐FTLB by following 30 patients initially diagnosed and carefully characterized in our clinic between 2019 and 2023. Through follow‐up (FU) assessments, we aimed to understand how symptom severity evolved over time and what factors might influence outcomes such as therapeutic interventions, comorbidities, secondary gain, exposure to social media, and patients’ acceptance of the diagnosis. Given that FND are an important and growing healthcare problem causing immense financial burden due to excessive testing and multiple consultations in specialists clinics,^27^, ^28^, ^29^, ^30^ identifying effective interventions and understanding the course of symptoms is essential for guiding clinical care and improving patients care.

## Methods

In this study, we provide FU data of a baseline data collection of a total of n = 53 patients with MSMI‐FTLB from our Tourette's outpatient clinic. Baseline data collection consisted of a thorough neuropsychiatric examination (done by KMV) and a detailed semi‐structured clinical and psychological interview (done by CF) performed between May 2019 and May 2023, aiming to establish the diagnosis of MSMI‐FTLB, to confirm or exclude a concurrent primary tic disorder, and included an interview (specifically designed for this study) with a detailed history of newly developed symptoms, focusing on type of onset (abrupt vs. gradual), symptom progression, triggering factors, suppressibility, distractibility, premonitory sensations, influencing factors, treatment, acceptance of diagnosis, and coincidence with the COVID‐19 pandemic. Baseline data of a subset of 32 patients have been published elsewhere.^3^

**Figure 1:**
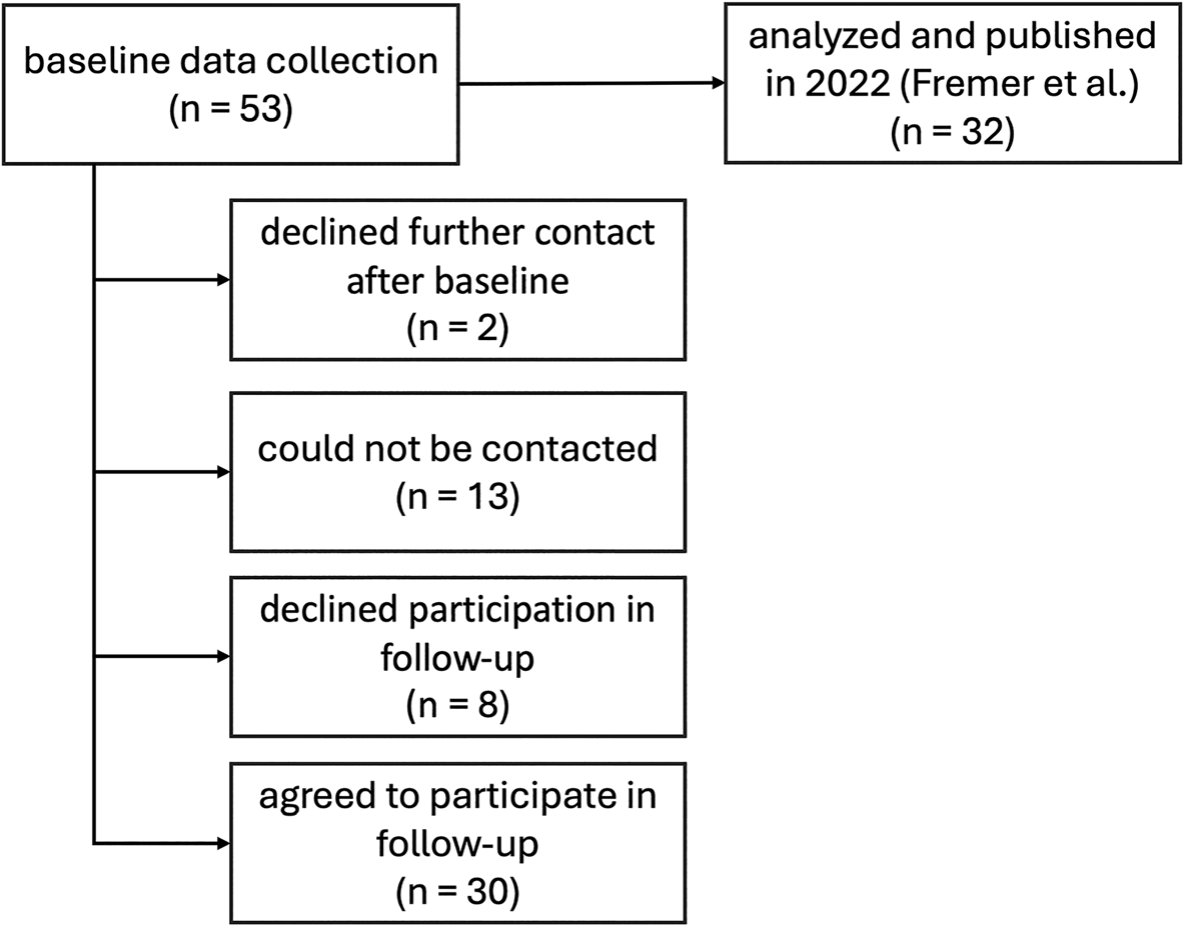
Flowchart of data collection process.

In some cases, the examination and baseline interview took place on the same day, while in others, they were separated by up to several months. Therefore, both time intervals were included in the analysis. Of n = 53 patients seen at baseline, n = 30 consented to participate in this FU study (done by LKH between January 2023 and March 2024). Using a Delphi method, we developed a comprehensive semi‐structured interview to explore the course of MSMI‐FTLB and to identify factors that may influence it, such as therapeutic interventions, changes in life circumstances, and the amount of time spent on social media. For a flow chart depicting the data collection process, see Figure 1.

Statistical analyses were conducted using JASP (JASP Team, 2025; version 0.19.3). The primary outcome variable was improvement of MSMI‐FTLB compared to baseline self‐assessed by the patients or caregiver as “much worse,” “minimally worse,” “no change,” “minimally improved,” “much improved,” and “remission” as well as quantified as “improvement in % (0–100%).” Due to non‐normal distribution of this variable, group comparisons were performed using the Mann–Whitney U test and the Kruskal‐Wallis test with Dunn's post‐hoc comparisons. Correlations were analyzed using Spearman's rank correlation coefficient. To account for multiple comparisons, *P*‐values were conservatively adjusted using Bonferroni correction. Detailed interview data were analyzed using inductive content analysis.^31^ Patients’ responses were categorized and, where applicable, grouped into superordinate categories. Comorbidities were assessed as “lifetime comorbidities,” relying on self‐reported diagnoses that were not clinically confirmed. This approach acknowledges that past diagnoses may indicate increased stress sensitivity, or underlying transdiagnostic neurobiological, psychological and/or social vulnerability. To quantify the burden of comorbidities, a comorbidity score was calculated based on the approach by Freeman et al^32^ and Spencer et al^33^ ranging from 0 (indicating “MSMI‐FTLB only” or “MSMI‐FTLB + TS” without any further psychiatric disorder) to 7 different psychiatric comorbidities (including other FNDs, depression, anxiety, obsessive‐compulsive disorder (OCD), post‐traumatic stress disorder (PTSD), personality disorder, and eating disorder). Although this method assumes equal weighting of all comorbidities, it provides a rough estimate of the overall complexity of a patient's condition.

To examine the content of psychotherapeutic interventions in detail, treatments were categorized as “(MSMI‐)FTLB‐specific” or “unspecific” based on the fact, whether—in the opinion of the patient/caregiver—(MSMI‐)FTLB were explicitly addressed or whether psychotherapy focused exclusively on more general aspects such as stress management or treatment of comorbid conditions. In addition, participants were asked, whether they felt psychotherapy helped to improve (MSMI‐)FTLB (“yes”/“no”).

Change in social media time was assessed based on the amount of screen time spent per day and whether daily usage had increased, decreased, or remained unchanged since baseline.

To assess the influence of patients’ social environment, secondary gain identified at baseline was re‐evaluated (still persistent, stopped). At baseline, patients were asked whether there were any aspects of their lives that had improved because of their symptoms. In addition to patients’ self‐reports, secondary gain was also rated as present if the interviewer identified evidence during the course of the interview (n = 5 patients). These factors included granting special privileges at home, school, or work (eg, permission to leave the classroom at will, exemption from household chores, or relief from more demanding tasks at work), receiving special attention from parents, friends, or partners through more affectionate and caring interactions, increased social recognition at school, or heightened attention on social media.

## Results

Of the 53 patients initially identified, n = 23 did not participate in the follow‐up (n = 11 male, n = 12 female; mean age at baseline = 18.65 years, SD = 4.69, range = 12–30). Within this group, n = 15 patients were diagnosed with MSMI‐FTLB and n = 8 with MSMI‐FTLB plus TS. There were no significant differences between participants and non‐participants regarding age at baseline, sex, and comorbid TS.

Thus, a total of n = 30 patients with MSMI‐FTLB were included (mean age at FU = 22.5 years, SD = 6 years, range = 14–57 years, median = 20 years); 18 participants (60%) were assigned female at birth; 15 (50%) identified as female, 3 (10%) as non‐binary, and 1 (3.3%) as a transgender man. Interviews were conducted face‐to‐face (n = 2, 6.7%), via video call (n = 27, 90%), or by phone (n = 1, 3.3%) depending on the patients’/families’ choice in order to perform as many FU interviews as possible. Interviews were conducted with n = 17 of the affected (56.7%) patients themselves, in n = 8 (26.7%) patients with both the patient and a caregiver (n = 7 mother, n = 1 primary caregiver), and with the caregiver only (in all cases the mother) in n = 5 (16.7%) cases.

Eleven (36.67%) patients fulfilled, in addition to MSMI‐FTLB, diagnostic criteria for TS. Further clinical characteristics are given in Table 1.

**TABLE 1:** Clinical characteristics in patients with MSMI‐FTLB including comparison between sex at birth

	All (N = 30)	Male (n = 12)	Female (n = 18)
Age at T2 (mean ± SD)	22.5 ± 11.6	20.1 ± 10.3	24.1 ± 12.4
Diagnosis			
MSMI‐FTLB (n, %)	19 (63.3%)	6 (50.0%)	13 (72.2%)
MSMI‐FTLB + TS (n, %)	11 (36.7%)	6 (50.0%)	5 (27.8%)
Co‐morbidities^a^			
None (n, %)	3 (10.0%)	2 (16.7%)	1 (5.6%)
Other FND (n, %)	13 (43.3%)	4 (33.3%)	9 (50.0%)
Depression (n, %)	16 (53.3%)	5 (41.7%)	11 (61.1%)
Anxiety (n, %)	16 (53.3%)	6 (50.0%)	10 (55.6%)
OCD/OCB (n, %)	10 (33.3%)	6 (50.0%)	4 (22.2%)
PTSD (n, %)	8 (26.7%)	1 (8.3%)	7 (38.9%)
ADHD (n, %)	6 (20.0%)	3 (25.0%)	3 (16.7%)
ASD (n, %)	6 (20.0%)	4 (33.3%)	2 (11.1%)
PD (n, %)	4 (13.3%)	1 (8.3%)	3 (16.7%)
Eating disorder (n, %)	3 (10.0%)	1 (8.3%)	2 (11.1%)
Psychosis (n, %)	1 (3.3%)	0 (0%)	1 (5.6%)
Comorbidity score (mean ± SD)	2.8 ± 1.8	2.6 ± 2.0	2.9 ± 1.7

Abbreviations: MSMI‐FTLB, mass social media‐induced functional tic‐like behavior; TS, Tourette's syndrome; FND, functional neurological disorder; OCD, obsessive compulsive disorder; OCB, obsessive compulsive behavior; PTSD, post‐traumatic stress disorder; ADHD, attention deficit/ hyperactivity disorder; ASD, autism spectrum disorder; PD, personality disorder. Due to limited statistical power, we focused on sex assigned at birth and did not report differences regarding gender.

^a^ Multiple answers possible.

The average time between first consultation due to MSMI‐FTLB at our TS clinic and FU was 29.23 months (SD = 8.97 months, range = 11–51 months, median = 28.5 months) and between baseline and FU 26.33 months (SD = 6.40 months, range = 11–43 months, median = 26.5 months).

**Figure 2:**
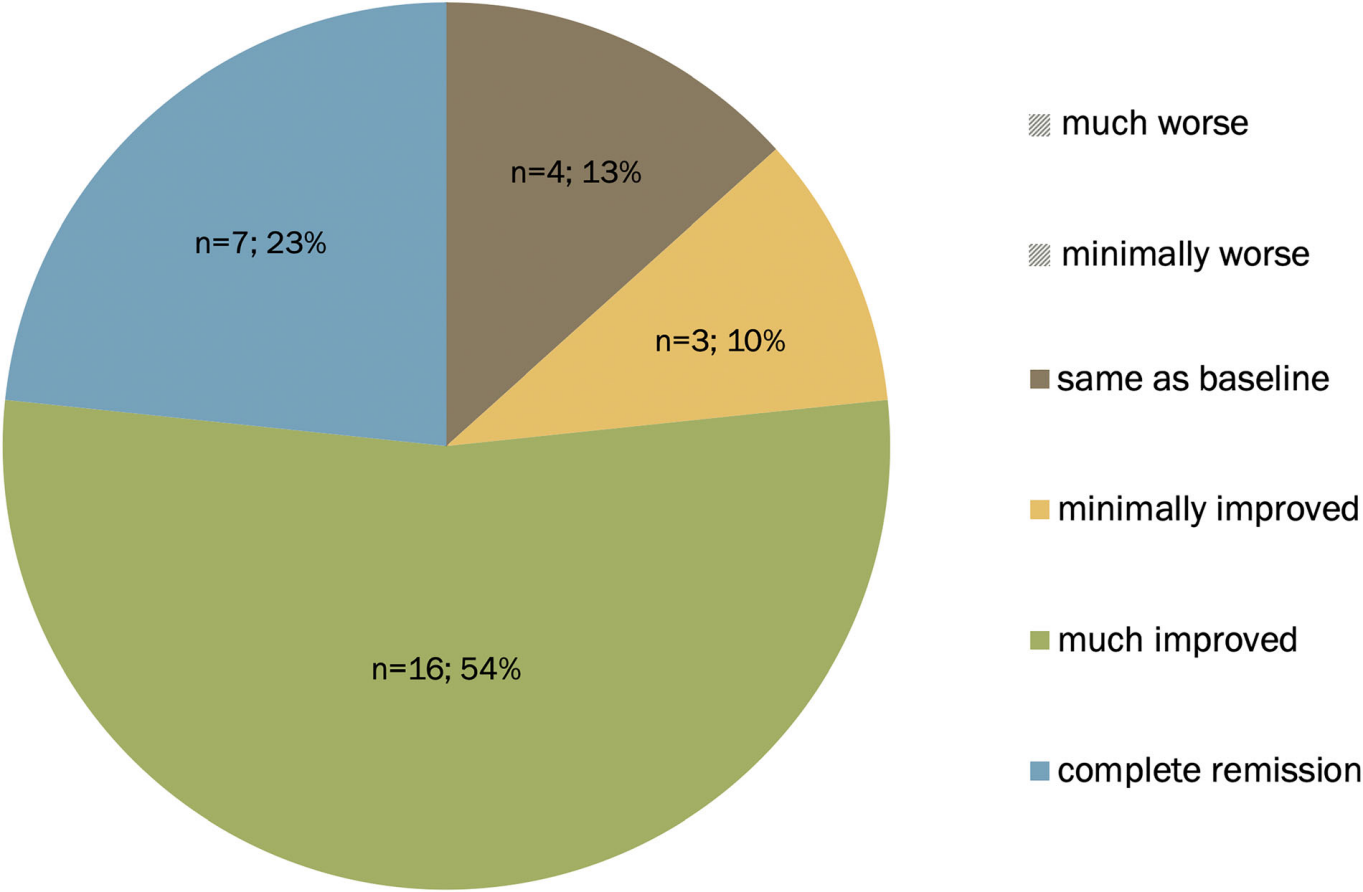
Subjective symptom severity at follow‐up compared to baseline assessment.

Compared to baseline, n = 7 (23%) reported “complete remission” of MSMI‐FTLB, n = 16 (54%) “much improvement,” n = 3 (10%) “minimal improvement,” n = 4 (13%) “no change,” and no participant reported “minimal worsening” or “much worsening” (Table 2, Fig. 2). Those who reported “minimal improvement” (n = 16) estimated improvement of 20.0% (SD = 0), while those who reported “much improvement” (n = 7) estimated improvement on average of 82.40% (SD = 16.95%, range = 45–99%, median = 90%). On average, subjective improvement was 68.03% (SD = 37.38%, range = 0–100%, median = 90%). Regarding clinical course of MSMI‐FTLB since baseline, n = 4 patients (13%) reported fluctuations, n = 17 (57%) gradual improvement, and n = 7 (23%) sudden improvement (Table 2).

**TABLE 2:** Course of MSMI‐FTLB and outcome at follow‐up including comparison between sex assigned at birth

	All (N = 30)	Male (n = 12)	Female (n = 18)
Course			
No change (n, %)	2 (6.7%)	1 (9.3%)	1 (5.6%)
Fluctuations (n, %)	4 (13.3%)	0 (0%)	4 (22.2%)
Slow improvement (n, %)	17 (56.7%)	8 (66.7%)	9 (50.0%)
Sudden improvement (n, %)	7 (23.3%)	3 (25.0%)	4 (22.2%)
Outcome			
Minimally worse (n, %)	0 (0.0%)	0 (0.0%)	0 (0.0%)
Much worse (n, %)	0 (0.0%)	0 (0.0%)	0 (0.0%)
Same as baseline (n, %)	4 (13.3%)	1 (8.3%)	3 (16.7%)
Minimally improved (n, %)	3 (10.0%)	1 (8.3%)	2 (11.1%)
Much improved (n, %)	16 (53.3%)	5 (41.7%)	11 (61.1%)
Complete remission (n, %)	7 (23.3%)	5 (41.7%)	2 (11.1%)
Symptom improvement in % (mean ± SD)	68.0 ± 37.4	80.7 ± 34.7	59.6 ± 37.6

*Note*: Due to limited statistical power, we focused on sex assigned at birth and did not report differences regarding gender.

We found a significant negative correlation between “subjective improvement (%)” and “time between MSMI‐FTLB onset and first consultation because of MSMI‐FTLB” (Spearman's correlation analysis, *ρ* = −0.438, *P* = 0.015). There was also a significant positive correlation between “subjective improvement” and “time between consultation and FU” (*ρ* = 0.403, *P* = 0.027). No significant correlation could be found between “subjective improvement” and “months between onset and FU” (*ρ* = 0.011, *P* = 0.954; Table 3).

**TABLE 3:** Duration of MSMI‐FTLB and correlation with subjective symptom improvement (0–100%)

Duration (months)	Descriptives			Improvement (%)	
	Range	Mean ± SD	Median	Spearman's *ρ*	*P*‐value
Months between onset and consultation^a^	1–40	13.73 ± 10.56	10.0	−0.438	0.015
Months between onset and follow‐up	28–59	42.97 ± 9.26	43.0	0.011	0.954
Months between consultation and follow‐up	11–51	29.23 ± 8.97	28.5	0.403	0.027
Months between baseline and follow‐up	11–43	26.33 ± 6.40	26.5	0.435	0.016

^a^ First consultation due to MSMI‐FTLB.

Statistical analysis for gender comparisons was limited due to the small sample size and the presence of only one transgender man. Methodologically, this individual was grouped with both the male and non‐binary categories to maintain statistical power. Kruskal‐Wallis tests (*P* = 0.110 when considering trans‐man as male; *P* = 0.090 when considered non‐binary) failed to show a significant impact of gender on improvement but a trend. However, a Mann–Whitney U test comparing assigned sex (female vs. male) regarding symptom improvement revealed a significant difference (*U* = 61, *P* = 0.047), with males showing better improvement, see Table 2.

**Figure 3:**
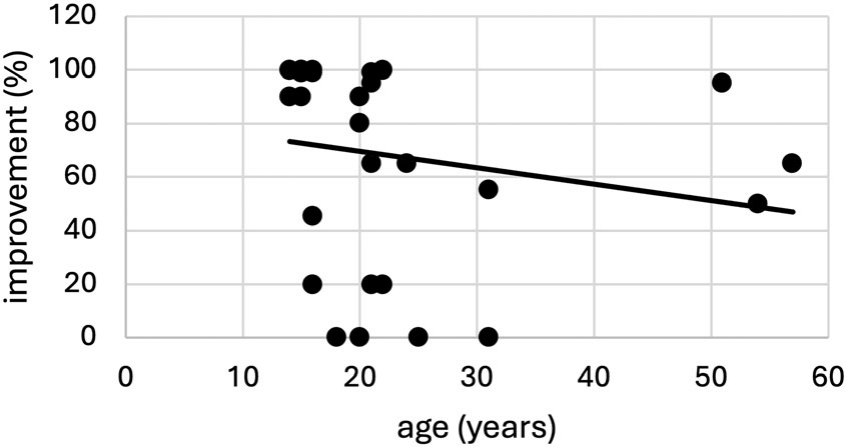
Correlation of age and degree of subjective symptom improvement (0–100%) as reported by patient/ caregiver.

There was a significant negative correlation between “age” and “subjective improvement” (*ρ* = −0.538, *P* = 0.002; Fig. 3) and a positive correlation between “age” and “number of comorbidities” (*ρ* = 0.56, *P* = 0.001).

**Figure 4:**
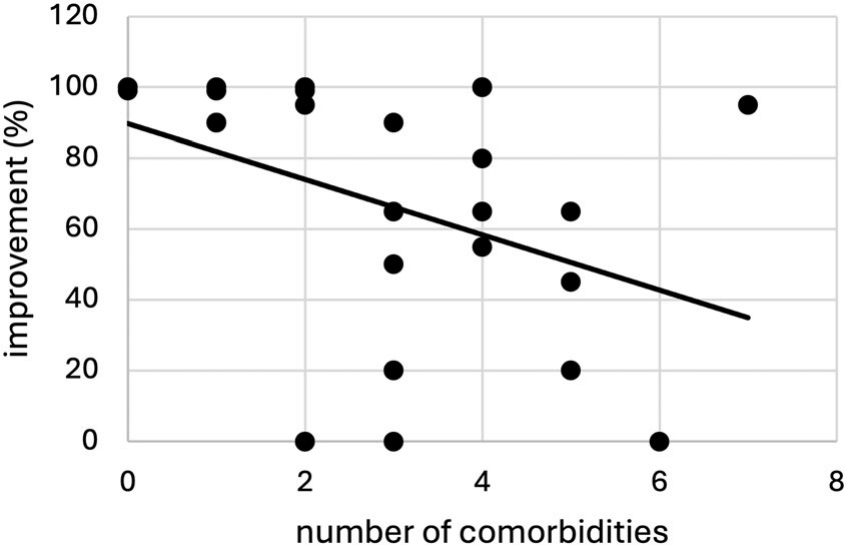
Correlation of comorbidity score (number of comorbidities) and subjective symptom improvement (0–100%) as reported by patient/ caregiver.

There was a significantly better outcome (improvement in %) in patients without any comorbidity (U = 11.5, *P* = 0.047). Correspondingly, higher comorbidity scores significantly correlated with worse outcomes (*ρ* = −0.458, *P* = 0.011; Fig. 4). When assessing each type of comorbidity separately, only depression had a detrimental effect on prognosis (U = 187.5, *P* = 0.002). There was no such effect for other types of FND (U = 135, *P* = 0.310). Comorbid TS also had no significant impact on subjective improvement (U = 108.5, *P* = 0.879).

Eleven patients (36.7%) reported that receiving the correct diagnosis had been helpful for their symptom improvement, whereas the remaining patients either observed no such association (50%) or were unable to tell whether knowing the diagnosis made a difference (13.3%). This group showed a borderline significantly better subjective improvement (mean = 85.73%, SD = 24.47) compared to those who did not perceive the diagnostic information as helpful (mean = 57.79%, SD = 40.21; *U* = 60.0, *P* = 0.056).

With regard to diagnostic acceptance, n = 23 patients (76.67%) expressed confidence that FTLB was the correct diagnosis. Six patients (20.0%) reported uncertainty, and only one participant (3.33%) explicitly rejected the diagnosis. However, diagnostic acceptance showed no significant impact on subjective symptom improvement (*U* = 39.0, *P* = 0.552).

Most patients (n = 26, 86.67%) received therapy: n = 24 (80.0%) outpatient psychotherapy, n = 9 (30%) inpatient psychotherapy, n = 4 (13.33%) body‐oriented therapy, and n = 5 (16.67%) other therapies such as hypnosis or family therapy. N = 15 patients (50%) received at least two different forms of treatment. Only very few patients were able to specify which therapeutic procedures (eg, cognitive behavioral therapy, CBT) they had undergone. Tic‐oriented interventions were reported in n = 7 cases; although patients could not always specify the exact method, these were most likely habit reversal training (HRT) in all cases. The interventions were consistently embedded within broader treatment approaches. While most patients reported no benefit for MSMI‐FTLB, two described that techniques such as redirecting or applying counter‐movements were helpful for selected motor symptoms. However, there was no significant difference in subjective symptom improvement (%) between patients who received therapy of any kind (mean = 70.81%, SD = 34.07%) and those who did not (mean = 50.0%, SD = 57.74%; U = 55.0, *P* = 0.878).

Among those who underwent therapy, n = 15 patients (57.56%) rated it as helpful, but this did not correspond to significantly greater improvement compared to those who found it unhelpful (U = 75.5, *P* = 0.491). Similarly, no significant differences in outcome were found between patients who received “MSMI‐FTLB‐specific” interventions and those who received unspecific treatment approaches (U = 41.5, *P* = 0.491; see Table 4).

**TABLE 4:** Improvement of MSMI‐FTLB (0–100%) and influencing factors

	n (% of n = 30)	Improvement in % (mean ± SD)
Any non‐pharmacological treatment		
No	4 (13.3%)	50.0 ± 57.7^a^
Yes	26 (86.7%)	70.8 ± 34.1
Non‐pharmacological treatment modality^b^		
Outpatient treatment	24 (80.0%)	71.7 ± 33.3
Inpatient treatment	9 (30.0%)	73.22 ± 38.1
Body oriented treatment	4 (13.3%)	76.3 ± 22.9
Other treatment	5 (16.7%)	69.2 ± 30.4
Therapeutic treatment focus		
MSMI‐FTLB‐specific	5 (16.7%)	85.4 ± 18.6
Unspecific	21 (70.0%)	67.3 ± 36.3
Pharmacotherapy		
None at baseline	11 (36.7%)	90.7 ± 23.8
All medication discontinued	6 (20.0%)	76.3 ± 39.7
Medication partially discontinued	3 (10.0%)	18.3 ± 31.8
Medication continued	4 (13.3%)	57.5 ± 31.2
New medication after baseline	6 (20.0%)	50.0 ± 35.1
Changes in daily social media‐time		
No change between baseline & follow‐up	11 (36.7%)	64.9 ± 40.4
Increased time	7 (23.3%)	65.0 ± 42.7
Decreased time	12 (40.0%)	72.7 ± 34.1
Secondary gain		
None at baseline	5 (16.7%)	69.0 ± 31.9
Stopped	16 (53.3%)	80.1 ± 32.1
Ongoing	9 (30.0%)	46.0 ± 42.3

MSMI‐FTLB, mass social media‐induced functional tic‐like behavior.

^a^ Out of the n = 4: n = 2 reported 100% improvement and n = 2 reported 0% improvement.

^b^ Multiple mentions possible.

At baseline, n = 19 patients (63.33%) received psychopharmacotherapy, which was completely discontinued at FU in n = 6 (20%), partially discontinued in n = 3 (10%) and continued in n = 4 (13.33%). In n = 6 patients (20%), additional psychopharmacological treatment was introduced on top of the baseline medication. Pharmacotherapy included both anti‐tic medication as well as medication for treatment of comorbidities, including antidepressants (selective serotonin reuptake inhibitors (SSRIs), serotonin–norepinephrine reuptake inhibitors (SNRIs), and tricyclic antidepressants (TCAs)), antipsychotics (typical and atypical), anxiolytics, stimulants, and cannabinoids. Among those n = 13 patients who received pharmacotherapy at FU, n = 11 (84.62%) reported no effect on MSMI‐FTLB, though they reported improvements in other symptoms such as mood, drive, sleep, and anxiety; n = 2 (15.38%) reported improvement in MSMI‐FTLB, both of whom were self‐medicating with cannabis.

No significant differences were found between changes in daily social media‐time (ie, “no change,” “decreased time,” “increased time”) and subjective outcome (H(df = 2) = 0.252, *P* = 0.882) (Table 4).

Patients with and without secondary gain at baseline did not differ significantly in the time from symptom onset to first consultation (Mann–Whitney‐U test, *P* = 0.303). The Kruskal–Wallis test comparing the groups “no secondary gain at baseline” (mean = 69.00%, SD = 31.90%), “secondary gain continued” (mean = 46.00%, SD = 42.25%), and “secondary gain discontinued” (mean = 80.13%, SD = 32.10%) revealed no significant overall difference (H^2^ = 4.282, *P* = 0.118). Given the small sample size, Dunn's post hoc comparisons were conducted for exploratory purposes. These revealed a significant difference between the groups “secondary gain continued” and “secondary gain discontinued” (*P* = 0.039) with the latter group showing a more favorable prognosis (Table 4).

## Discussion

We present the results of one of the few studies on long‐term FU in patients with FTLB. To the best of our knowledge, this is the first study to investigate prognosis in the specific subset of MSMI‐FTLB. Our data suggest that MSMI‐FTLB (with or without comorbid TS) generally has a favorable prognosis, as most patients experienced a symptom improvement over time. Factors related to favorable outcome included younger age, male sex, early diagnosis, discontinuation of secondary gain, less psychiatric comorbidities, and absence of depression.

Previous research shows that the course of MSI depends on its clinical subtype and context. In mass anxiety hysteria, symptoms spread rapidly but usually remit once groups are separated, whereas mass motor hysteria tends to evolve more gradually and may persist for weeks or months, particularly in socially tense, isolated, or highly regulated settings where stressors remain unresolved.^34^, ^35^, ^36^ While mass motor hysteria may already follow a more gradual and prolonged course, in our cohort, MSMI‐FTLB presented in some cases as persistent and clinically complex, whereas in others symptoms remitted quickly and spontaneously. Overall, once the correct diagnosis is established, the prognosis is generally favorable. Longer or more complicated courses may be explained by continuous exposure and reinforcement through social media, with prominent index patients providing repeated opportunities for disease modeling. Such behaviors may become integrated into patients’ identity and further stabilized by ongoing social reinforcement and psychiatric comorbidities, thereby reducing the likelihood of remission.

Our finding of a favorable prognosis in MSMI‐FTLB is also in line with previous reports of FTLB,^23^, ^25^, ^37^, ^38^, ^39^ including prospective FU studies after 6 and 12 months in 29 and 83 patients, respectively, using the Yale Global Tic Severity Scale (YGTSS).^40^ In a study in 56 patients, 79% improved at FU after 17 months.^38^ Similarly, Ducroizet et al reported a favorable outcome in 65% of the 43 patients after a mean of 2.6 years,^39^ while Prato et al, in a 12‐month follow‐up of 243 patients, observed an overall, but statistically non‐significant, improvement based on YGTSS scores.^23^

Nearly 80% of our patients reported MSMI‐FTLB as “improved” or in “complete remission.” While onset of FTLB is mainly abrupt, improvement was reported by the majority of patients as gradual. However, one fourth of patients experienced sudden improvement. Individual variability in improvement indicates that not all patients have the same prognosis. These findings align with previous research on FMD, which also highlights a heterogeneous course and varying responses to intervention.^41^

As suggested by previous studies,^25^, ^37^ we also found that younger age is related overall to better prognosis which could be related to lower number of comorbidities, but also to easier learning processes at younger age, different coping mechanisms and protective social factors across the lifespan.^42^, ^43^ We found a significant negative correlation between the duration between onset and consultation on the one hand and symptom improvement on the other. Similar results have been observed in studies on other FMD, where shorter symptom duration and access to specialized clinics has been associated with better recovery rates.^44^, ^45^ This highlights the need for improved recognition and earlier intervention in patients of (MSMI‐)FTLB, reinforcing the importance of early diagnosis and treatment to prevent symptom persistence and chronic progression.

Another key finding was that a higher comorbidity score—particularly the presence of depression—was associated with poorer outcomes. The particularly negative impact of depression may be related to its impact on coping mechanisms, which may contribute to symptom persistence in FTLB,^46^ which is especially relevant considering that depression and anxiety are among the most common comorbidities in patients with FTLB.^3^, ^15^, ^47^ This finding also underscores the importance of treatment of psychiatric comorbidities, and in particular depression.

In contrast to previous studies,^25^ we did not find evidence for a negative impact of other psychiatric comorbidities, such as additional FND, on symptom improvement. This also applied to TS, which was present in over one third of patients, consistent with earlier reports,^48^ but showed no influence on overall prognosis.

It has been repeatedly reported that prevalence of sex and gender minority groups is significantly higher in patients with FTLB compared to healthy controls and patients with TS.^38^, ^49^ We did not find any difference in disease prognosis between cis‐ and gender diverse individuals. In terms of sex differences, however, male patients showed significantly higher improvement than female patients. This may have biological, psychological, or social underpinnings, such as sex‐related differences in symptom processing, hormonal differences, or societal expectations regarding coping strategies.^49^, ^50^, ^51^ This stays in line with previous reports in FND, which has demonstrated higher prevalence and clinical symptomatology of FND in females, suggesting that the sex‐related differences observed in our cohort may reflect broader mechanisms beyond movement disorders alone.^50^

Despite the high proportion of patients who received psychotherapy, no significant differences in symptom improvement were observed between those who underwent treatment and those who did not. Furthermore, among those who received therapy, neither the perceived helpfulness of the intervention nor the specificity of the therapeutic approach was associated with a more favorable outcome. These findings may appear counterintuitive, but they align with previous studies,^25^, ^37^, ^38^ suggesting that the effectiveness of psychotherapy is highly variable and dependent on individual factors such as patient engagement and therapeutic alliance.

Similar to recent studies in FMD, we found no improvement of MSMI‐FTLB by pharmacological treatment.^52^ Interestingly, in our study, only those two patients using cannabis reported an improvement in MSMI‐FTLB. While this might indicate a potential therapeutic role of the endocannabinoid system in FTLB, in can also be speculated that cannabis reduces anxiety and stress, increases relaxation, or only has a placebo effect. However, improvement of FTLB after use of cannabis has also been reported in a case series in five patients.^53^

Since in our sample, a clear association between social media exposure and symptom onset could be demonstrated, we were particularly interested in the impact of social media consumption on the prognosis of MSMI‐FTLB.^3^, ^5^ Contrary to our expectation, a reduction in social media use had no direct impact on prognosis, suggesting that social media exposure may function more as a triggering than a maintaining factor. It would have been of interest to examine whether changes in the type of content consumed influenced outcomes; however, this aspect was not assessed in the present study.

Since in our baseline study we identified secondary gain due to MSMI‐FTLB in a substantial number of patients,^3^ in this FU study we were able to reassess this potentially influencing factor and found a trend towards better prognosis in patients who discontinued secondary gains. This finding aligns with previous research on FND, where attention to symptoms and secondary gain have been identified as contributing factors to symptom persistence, indicating that such mechanisms are relevant across different functional syndromes, including FTLB.^54^

Despite multiple strengths of our study, several limitations must be acknowledged. Firstly, some interviews were conducted exclusively with the patients’ mothers or in the presence of caregivers, which may have introduced response bias due to social desirability or caregiver influence. Secondly, the time between the initial consultation/baseline and FU varied considerably, which may have led to recall bias and differences in natural symptom fluctuations. Thirdly, the sample size is relatively small. However, the study focused on a highly specific subtype of FTLB, MSMI‐FTLB, from one single center. Fourthly, the primary outcome variable relies on subjective self‐reporting, which may not always align with clinical assessments. Nevertheless, subjective improvement is a key component of patient‐centered care, and the observed correlations with other symptom measures suggest meaningful real‐world relevance. Finally, psychiatric comorbidities were assessed only in terms of lifetime prevalence, without standardized diagnostic tools or differentiation by current symptom severity, which may have limited the precision of comorbidity‐related analyses. Future studies should also incorporate qualitative data to better capture patients’ perspectives on useful interventions.

## Author Roles

(1) Research project: A. Conception, B. Organization, C. Execution. (2) Statistical Analysis: A. Design, B. Execution, C. Review and Critique. (3) Manuscript Preparation: A. Writing of the first draft, B. Review and Critique. All authors contributed to manuscript revision, read, and approved the submitted version.

L.K.H.: 1A, 1B, 1C, 2A, 2B, 3A, 3B.

S.S.: 1A, 2A, 2B, 2C, 3B.

C.F.: 1A, 1B, 1C, 2C, 3B.

C.S.K.: 1A, 1B, 2C, 3B.

N.S.: 2C, 3A, 3B.

K.M.V.: 1A, 1B, 1C, 2C, 3B.

## Disclosures

**Ethical Compliance Statement:** The study was reviewed and approved by Local Ethics Committee at Hannover Medical School (No. 8995_BO_S_2020). Written informed consent to participate in this study was provided by the participants and their legal guardian/ next of kin. We confirm that we have read the Journal's position on issues involved in ethical publication and affirm that this work is consistent with those guidelines.

**Funding Sources and Conflict of Interest:** No specific funding was received for this work. The authors declare that there are no conflicts of interest relevant to this work.

**Financial Disclosures for the previous 12 months:** KM‐V has received financial or material research support from DFG: GZ MU 1527/3–1 and GZ MU 1527/3–2, and Almirall Hermal GmbH. She has received consultant fees and other honoraria from AlphaSights Ltd., Aurora, Canopy, Cansativa, Canymed, DHMS Direct Health Medical Services Ltd./ Wellster Healthtech Group, Emalex, Neuraxpharm, Renafan, Sanity Group, Synendos Therapeutics AG, Takeda, and Tetrapharm. She is an advisory/ scientific board member for Branchenverband Cannabiswirtschaft e.V. (BvCW), Sanity Group, Synendos Therapeutics AG, and Therapix Biosciences Ltd. She has received speaker's fees from Ärztekammer Niedersachsen, Bundesverband der pharmazeutischen Cannabinoidunternehmen (BPC), Cogitando GmbH, diaplan GmbH, FomF GmbH, Grow, Laleto GmbH, Landesamt für Soziales, Jugend und Versorgung Mainz, Noema, streamedup! GmbH, VBG—Unfallversicherung Hamburg, Universitätsklinikum Hamburg‐Eppendorf, Universitätsklinikum Münster, and WeCann. She has received royalties from Elsevier, Medizinisch Wissenschaftliche Verlagsgesellschaft Berlin, and Kohlhammer. She is an associate editor for “Cannabis and Cannabinoid Research.” She is an Editorial Board Member of “Medical Cannabis and Cannabinoids” and “MDPI‐Reports” and a Scientific board member for “Zeitschrift für Allgemeinmedizin.”

NS is an advisory/ scientific board member for Cosma S.A. She served as a Guest Editor for *MDPI* including *Healthcare and International Journal of Molecular Sciences* and *BMC Complementary Medicine and Therapies* for the Special Issue “Advances in cannabis and cannabinoid research” and *Frontiers in Psychiatry*.

The remaining authors declare that there are no additional disclosures to report.

## Data Availability

The data that support the findings of this study are available on request from the corresponding author. The data are not publicly available due to privacy or ethical restrictions.
